# The relationship between the EAT-Lancet dietary pattern and risk of cardiovascular events in patients with established cardiovascular disease

**DOI:** 10.1007/s00394-025-03754-2

**Published:** 2025-12-01

**Authors:** Lukas L. F. Hoes, Chiara Colizzi, Yvonne T. van der Schouw, Johanna M. Geleijnse, Jannick A. N. Dorresteijn, Manon G. van der Meer, Martin Teraa, Frank L. J. Visseren, Charlotte Koopal

**Affiliations:** 1 Department of Vascular Medicine, University Medical Center Utrecht, Utrecht University, Utrecht, The Netherlands; 2 Julius Center for Health Sciences and Primary Care, University Medical Center Utrecht, Utrecht University, Utrecht, The Netherlands; 3 Division of Human Nutrition and Health, Wageningen University and Research, Wageningen, Netherlands; 4 Department of Cardiology, University Medical Center Utrecht, Utrecht University, Utrecht, The Netherlands; 5 Department of Vascular Surgery, University Medical Center Utrecht, Utrecht University, Utrecht, The Netherlands

**Keywords:** Secondary prevention, Plant-based diet, Vegetarian diet, Coronary artery disease, Cerebrovascular accident, Lifestyle

## Abstract

**Purpose:**

To reduce the risk of recurrent cardiovascular events in patients with established cardiovascular disease (CVD), guidelines recommend adopting a more plant-based diet. The EAT-Lancet diet, which focuses on plant-based foods, is associated with a lower risk of CVD in apparently healthy people. However, the relationship in patients with established CVD is unclear. Therefore, the aim of this research was to quantify the relationship between the EAT-Lancet Healthy Reference Diet (HRD) and risk of recurrent CVD in patients with established CVD.

**Methods:**

Patients with established CVD from the Utrecht Cardiovascular Cohort-Second Manifestations of ARTerial disease were studied. Dietary intake was measured using a food frequency questionnaire and the relationship between the EAT-Lancet HRD (score from 0 to 140 points) and non-fatal myocardial infarction and stroke was assessed using Cox regression adjusted for age, sex, educational background, lifestyle factors, and energy intake.

**Results:**

During a follow-up of 24,212 person-years 209 non-fatal vascular events occurred. The median score for the EAT-Lancet HRD was 57 out of 140 points (IQR: 41–68). After adjustment for confounders, a diet more in line with the EAT-Lancet HRD was associated with a lower risk of non-fatal vascular events (HR 0.87 (95% CI 0.79–0.96) per 10-point increase); stroke (HR 0.76 (95% CI 0.63–0.91) per 10-point increase); and a trend towards lower risk of myocardial infarction (HR 0.90 (95% CI 0.81–1.02) per 10-point increase).

**Conclusion:**

In patients with established CVD, a dietary pattern more in line with the EAT-Lancet HRD is associated with a lower risk of non-fatal stroke and myocardial infarction.

**Supplementary Information:**

The online version contains supplementary material available at 10.1007/s00394-025-03754-2.

## Introduction

Patients with established cardiovascular disease (CVD) are at high or very high risk of new cardiovascular events [1]. Guidelines recommend adopting a more plant-based dietary pattern to improve cardiovascular risk profile and potentially reduce CVD risk [1, 2]. The EAT-Lancet diet is such a dietary pattern. Compared to the CVD guidelines, the EAT-Lancet diet features more specific recommendations regarding the intake of plant-based foods, such as soy and legumes and recommends further reductions of animal-based foods, such as red meat. The EAT-Lancet diet was designed to be both healthy and sustainable within planetary boundaries. The diet consists primarily of fruits, vegetables, whole grains, legumes, nuts, and unsaturated (plant-based) oils [3]. Additionally, the dietary pattern consists of small amounts of seafood and poultry and minimal amounts of red meat, processed meat, added sugar, starchy vegetables and refined grains [3]. In apparently healthy people, cross-sectional studies have shown a relation between higher adherence to the EAT-Lancet dietary pattern and lower systolic blood pressure (SBP) and low-density lipoprotein cholesterol (LDL-c) [4]. Furthermore, in apparently healthy people high compared to low adherence to the EAT-Lancet diet is associated with a 12% lower risk of coronary artery disease (CAD) and an 11% lower risk of stroke [5]. However, there is no evidence regarding the relationship between the EAT-Lancet diet and CVD risk in patients with established CVD, a patient group that usually already receives cholesterol- and blood pressure-lowering therapies. Dietary habits partly influence CVD risk through their effect on risk factors such as lipids, SBP, body weight and diabetes mellitus [1]. Therefore, the effect of diet might be different in patients with established CVD. This study aims to quantify the relationship between the EAT-Lancet diet and CVD risk in patients with established CVD.

## Methods

### Study population

Patients with established CVD from the Utrecht Cardiovascular Cohort-Second Manifestations of ARTerial disease cohort (UCC-SMART cohort) were studied. A complete overview of the cohort and the definitions used is available in the UCC-SMART cohort profile [6]. In short, UCC-SMART is an ongoing prospective cohort including patients with established CVD or the presence of severe cardiovascular risk factors such as hypercholesterolemia or (treatment resistant) hypertension. Inclusion started in September 1996 and is ongoing with on average 300–400 CVD patients being included annually. The study has been approved by the ethical review board of the University Medical Center Utrecht. Written informed consent is retrieved from all patients, before inclusion in the study. For the present study, only patients with established CVD (coronary artery disease (CAD), peripheral artery disease (PAD), abdominal aortic aneurysm (AAA) and/or cerebrovascular disease (CeVD)) were included.

### Dietary intake assessment

In 2022, an invitation to complete a food frequency questionnaire (FFQ) was sent out to all living participants of the UCC-SMART cohort, who were in active follow-up [7]. Out of 10,072 patients, 4,496 responded, yielding a response rate of 45%. The FFQ utilized was the FFQ-NL 1.0, which was specifically developed to capture dietary intake in the Dutch population [8]. This questionnaire has been validated against 24-h recalls and both urine and serum biomarkers, with validity ranging from acceptable to good [8]. Based on the reported dietary intake, dietary energy, macro- and micronutrient intake was calculated using the Dutch Food Composition Database edition 2010 [9]. Patients who reported a caloric intake below 500 kcal/day or above 3500 kcal/day were excluded from the analysis (N = 487, Fig. S1). A total of 2,685 patients who filled out the FFQ had CVD at baseline. However, baseline characteristics of CVD patients included in the year 2023 were not yet systematically available (N = 132, Fig. S1), resulting in 2553 patients with CVD in the final dataset.

### Collection of baseline characteristics

At baseline, patients undergo a vascular screening, which includes amongst others, a questionnaire on medical history, laboratory measurements of risk factors and ultrasound of the carotid arteries and abdominal aorta [6]. In short, the standardized questionnaire collects data on current use of medication and medical history. Serum cholesterol, glycated hemoglobin, creatinine and C-reactive protein (CRP) are measured using venous blood collected on the morning of the vascular screening, after having fasted for at least 8 hours. Additionally, patients undergo a physical examination, including measurement of height and weight, while wearing light clothes and no shoes. Office blood pressure is measured in a standardized manner. Measurements are performed unattended at both upper arms with the patient in a supine position. Three measurements are taken at an interval of 30 s. Mean blood pressure is then calculated using the measurements on the arm with the highest blood pressure. A detailed description of the UCC-SMART-cohort and all study procedures has previously been published elsewhere. [6]

### Calculation of the EAT-Lancet score

Compliance with the EAT-Lancet Diet was quantified using the previously published EAT-Lancet Healthy Reference Diet score [5]. This score has been used to assess the relationship between the EAT-Lancet HRD and risk of CVD in the general population and was adapted to local Dutch dietary habits. The score consists of 14 proportional scores ranging from 0 to 10 for 14 different dietary components (Table S1), resulting in a total score of 0 to 140. Each component is categorized as either an adequacy, optimum or moderation component (Table S1). Adequacy components are fruits, vegetables, whole grains, legumes and soy and a higher intake results in a higher score, with a maximum of 10 points for each component. Moderation components are red meat (pork, beef, lamb) and sweeteners and a higher intake of these components leads to a lower score. Optimum components consisted of food groups with a high nutritional value, but with potential adverse health effects if eaten in large quantities. Until a certain threshold, a higher intake results in a higher score for these components. Once this optimum threshold is passed, the score declines. Optimum components are potatoes, dairy, chicken, eggs, fish, and nuts (Table S1). In the original EAT-Lancet HRD score by Colizzi et al. fats were scored by calculating the ratio of unsaturated to saturated oil intake [5]. In the FFQ-NL, intake of added oils primarily focuses on unsaturated oils. Therefore, in this article fats are scored based on absolute intake of unsaturated oils (olive oil, liquid baking products and margarine, Table S1). Unsaturated oil was scored as an optimum component, using 40–80 g of added unsaturated oils as optimum, which is the target for unsaturated oils in the original EAT-Lancet report (Table S1) [3].

Scores of all food components were calculated based on the intake reported in the FFQ. For pasta and rice no information was available regarding grain type (whole grain or refined grain). Therefore, pasta and rice were not included in the total intake of whole-grain products. In the Netherlands, bread contributes to 17% of energy intake, while rice and pasta contribute to about 3% [10]. An unknown percentage from this 3E% for rice and pasta is whole grain. The contribution of whole grain rice and pasta to the total diet is therefore limited. Dry weight of pulses, rice and pasta was calculated by dividing cooked weight by a factor 2.5, 2.5 and 1.83 respectively [11, 12]. Scores (0–10) of all 14 components were summed to derive the EAT-Lancet HRD score. Additionally, an energy-adjusted score was calculated using the residuals method to account for differences based on energy-intake [13].

### Outcomes

The primary outcome was non-fatal vascular events, a composite of non-fatal myocardial infarction and non-fatal stroke (ischemic and hemorrhagic). Data on fatal events were not available given that the present study uses data from patients who filled out the FFQ in 2022 and therefore had not experienced a fatal event. The determination of vascular endpoints in the UCC-SMART cohort has been previously described [6]. In brief, endpoints are assessed by an endpoint committee consisting of three physicians through consensus [6]. Stroke was defined as at least 24 hours of clinical signs in combination with a Rankin scale score of 1 or more. Stroke type is determined using imaging (computed tomography or magnetic resonance imaging) or in surgery. Myocardial infarction is defined as chest pain (> 30 min) in combination with elevated cardiac enzymes and/or documented ECG changes [6].

### Data analyses

Baseline characteristics are described using mean (standard deviation), median [25–75th percentile] or absolute number (percentage). Cox proportional hazard regression was used to assess the relationship between the EAT-Lancet HRD and non-fatal vascular events, a composite of myocardial infarction and stroke. Three Cox regression models were used. Model 1 adjusted for age and sex. Model 2 (main model) additionally adjusted for level of education, physical activity, alcohol intake, smoking status, number of packyears, year of inclusion and energy intake. Model 3 (exploratory model) additionally adjusted for possible intermediates, i.e. body mass index, non-high-density-lipoprotein cholesterol (non-HDL-c), systolic blood pressure (SBP), glycated haemoglobin (HbA1c), estimated glomerular filtration rate (eGFR), use of statins and number of antihypertensives. The proportional hazard assumption was checked visually by plotting the Schoenfeld residuals. Potential non-linearity was assessed by adding splines with 3 and 4 knots to model 2. Interaction in subgroups was assessed by including an interaction term between the EAT-Lancet HRD score (continuous) and each subgroup (continuous for age, dichotomous for all other subgroups) to the model on at the time. Subgroup analyses were performed for age, sex, diagnoses of CAD, CeVD, PAD/AAA or T2D and in strata of risk factor levels (BMI, eGFR, SBP, HbA1c, non-HDL-c).

Sensitivity analyses were conducted including only patients who filled out the FFQ within nine years of inclusion. Nine years was chosen using the results from the control group of a trial in obese patients undergoing bariatric surgery, which showed that after an initial improvement after inclusion, dietary intake remained relatively stable during an additional nine years of follow-up [14]. Furthermore, the impact of excluding each of the 14 components from the EAT-Lancet HRD score one at a time was assessed. For this purpose, 14 separate EAT-Lancet HRD scores were calculated, each with one component omitted. As a result, these scores consisted of 13 components and ranged from 0 to 130 instead of 140 points. Lastly, it was assessed whether the associations differed when the energy-adjusted EAT-Lancet HRD score was used. In the main analysis, the relationship between EAT-Lancet HRD score and CVD was adjusted for energy-intake in the main Cox model (model 2). In the sensitivity analysis, the residual method was used to calculate an energy-adjusted score which replaced the unadjusted score from the main model [13].

## Results

### Patient characteristics

A total of 2553 patients with CVD filled out the food frequency questionnaire and were included in the study (Fig. S1). Mean age was 59 ± 9 years, 22% of patients were female, 42% had a high level of education and 29% reported never smoking at baseline. Mean body mass index was 26.7 ± 3.9 kg/m^2^ (Table 1). 1625 patients (64%) were diagnosed with CAD at baseline and 215 (8%) had CVD at more than one location. 11% of patients were diagnosed with T2D in addition to CVD (Table 1). 79% of patients used statins and patients used an average of 2.5 ± 1.1 medications with an antihypertensive effect. On the other hand, among CVD patients who did not fill out the FFQ 26% had a high education level and 24% reported never smoking at baseline (Table S8).

**Table 1:** Baseline characteristics of patients, stratified by adherence to the EAT-Lancet healthy reference diet

	Overall	EAT-Lancet healthy reference diet		
	(N = 2553)	1st tertile (N = 851)	2nd tertile (N = 851)	3rd tertile (N = 851)
Age (years)	59 ± 9	59 ± 9	59 ± 10	59 ± 9
Sex (female)	557 (22)	182 (21)	181 (21)	194 (23)
*Level of education*				
Low	479 (19)	207 (24)	172 (20)	100 (12)
Medium	1006 (39)	354 (42)	332 (39)	320 (38)
High	1068 (42)	290 (34)	347 (41)	431 (51)
*Alcohol consumption (units/week)*				
0–10	1893 (74)	641 (75)	622 (73)	630 (74)
11–20	463 (18)	142 (17)	170 (20)	151 (18)
> 20	197 (8)	68 (8)	59 (7)	70 (8)
*Smoking status*				
Never	743 (29)	221 (26)	235 (28)	287 (34)
Current	500 (20)	208 (24)	168 (20)	124 (15)
Former	1310 (51)	422 (50)	448 (53)	440 (52)
Packyears	9 [0–24]	11 [0–27]	10 [0–24]	6 [0–20]
*Physical activity (METh/week)*				
1st tertile	851 (33)	318 (37)	301 (35)	232 (27)
2nd tertile	851 (33)	272 (32)	272 (32)	307 (36)
3rd tertile	851 (33)	261 (31)	278 (33)	312 (37)
*History of CVD*				
CAD	1625 (64)	510 (60)	547 (64)	568 (67)
CeVD	502 (20)	184 (22)	157 (18)	161 (19)
PAD and/or AAA	211 (8)	78 (9)	74 (9)	59 (7)
> 1 Location	215 (8)	79 (9)	73 (9)	63 (7)
Hypertension	1321 (52)	425 (50)	447 (53)	449 (53)
Type 2 diabetes	276 (11)	95 (11)	96 (11)	85 (10)
BMI (kg/m^2^)	26.7 ± 3.9	27.1 ± 4.1	26.7 ± 3.6	26.4 ± 4.1
SBP (mmHg)	134 ± 18	135 ± 20	134 ± 17	133 ± 18
Glycated hemoglobin (%)	5.6 [5.4–5.8]	5.6 [5.4–5.9]	5.6 [5.4–5.9]	5.5 [5.4–5.8]
Non-HDL-c (mmol/L)	3.0 [2.5–3.8]	3.1 [2.5–3.9]	3.0 [2.5–3.8]	2.9 [2.3–3.6]
eGFR (mL/min/1.73m^2^)	91 [78–100]	89 [76–99]	90 [78–100]	93 [82–101]
C-reactive protein (mg/L)	1.5 [0.8–3.1]	1.6 [0.9–3.4]	1.6 [0.8–3.0]	1.4 [0.8–3.0]
NLR	1.9 [1.5–2.5]	1.9 [1.5–2.5]	1.9 [1.5–2.5]	1.9 [1.4–2.5]
Number of anti-hypertensives	2.5 (1.1)	2.5 (1.1)	2.5 (1.1)	2.5 (1.1)
Statin usage (%)	2014 (79)	657 (77)	666 (78)	691 (81)

Data are presented as mean (standard deviation), median [interquartile range] or count (percentage). CVD = (cardio)vascular disease. CeVD = cerebrovascular disease. PAD = peripheral artery Disease. AAA = abdominal aortic aneurysm. BMI = body mass index. SBP = systolic blood pressure. Non-HDL-c = non-high-density lipoprotein cholesterol. eGFR = estimated glomerular filtration rate calculated using creatinine [1]. NLR = neutrophil-to-lymphocyte ratio

### Alignment of reported diet with the EAT-Lancet HRD

**Fig. 1:**
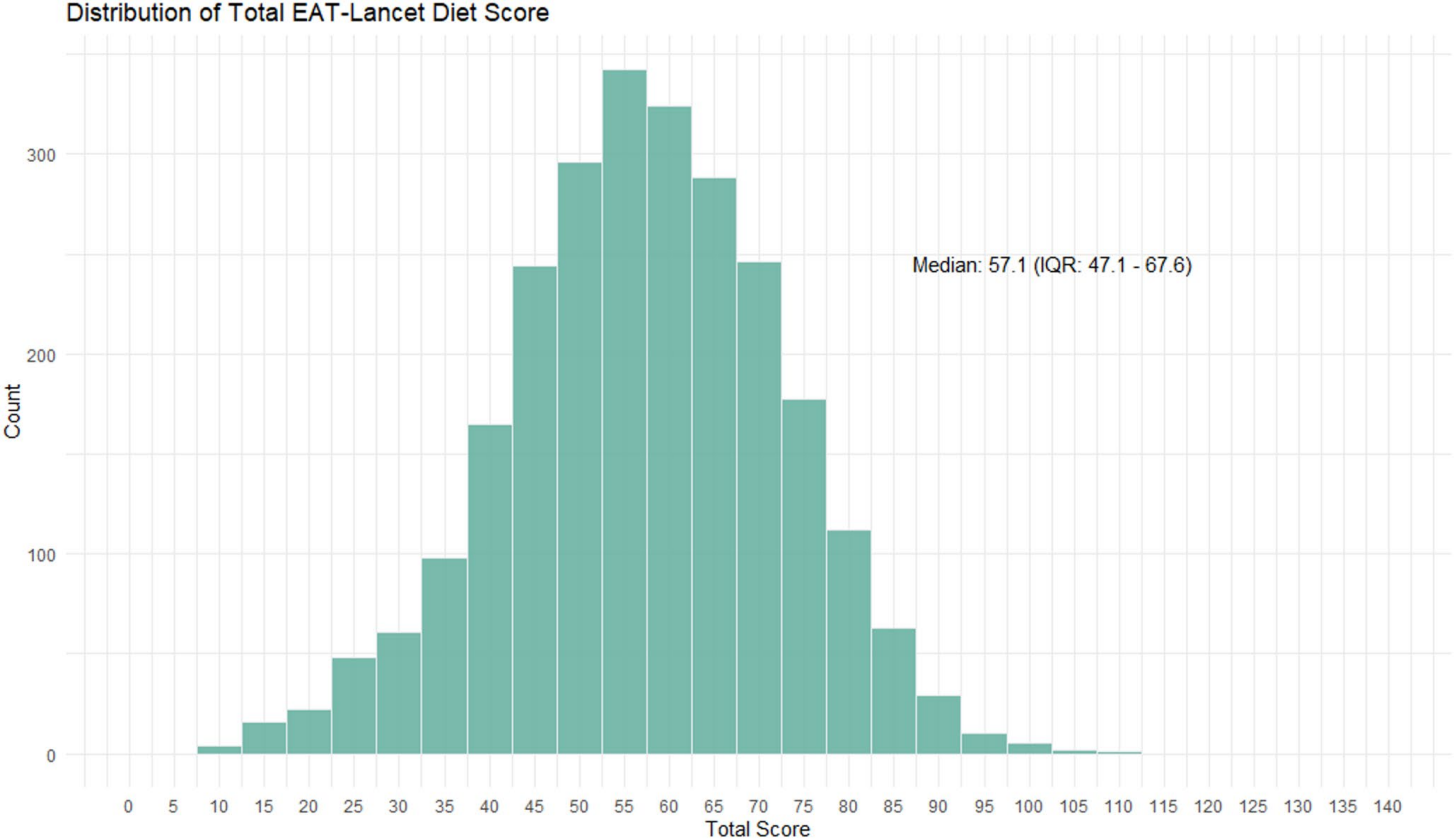
Distribution of the EAT-Lancet Healthy Reference Diet score in the study population. The score ranges from 0 to 140. Bins increase in steps of 5 points. IQR = interquartile range

**Fig. 2:**
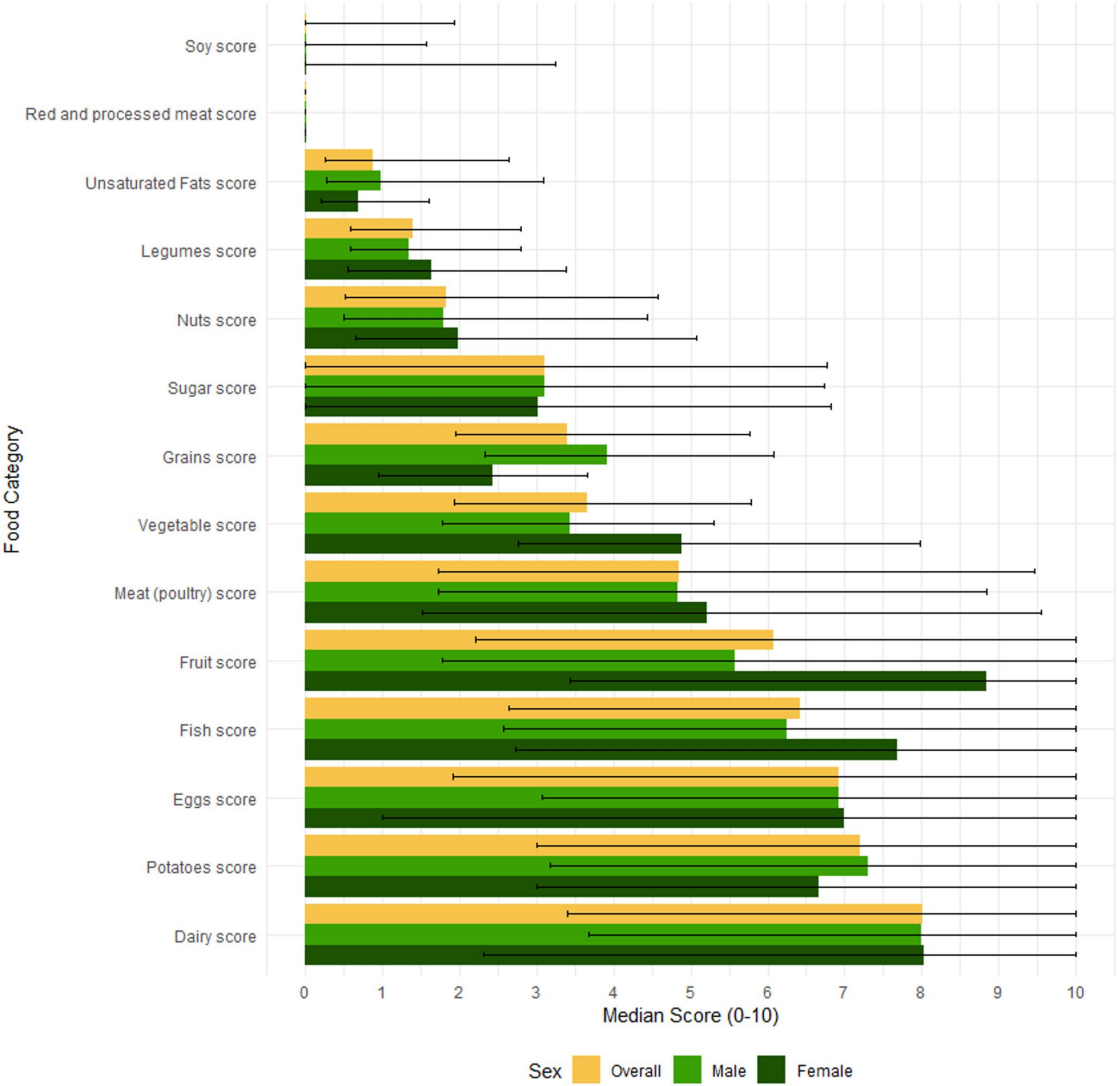
Median score with interquartile range per food category of the EAT-Lancet Healthy Reference Diet. The maximum score for each food category is 10. For whole grains, vegetables, fruits, legumes and soy (adequacy components), a higher intake resulted in a higher score. For potatoes, dairy, chicken, eggs, fish, nuts and unsaturated oils (optimum components), an intake below or above an optimum resulted in a lower score. A higher intake of red meat and sugar from sweeteners resulted in a lower score (moderation components). A detailed overview of the calculation for each subgroup is provided in Table S2

The median score for the EAT-Lancet HRD was 57.1 out of a maximum of 140 points (IQR: 47.1–67.6, Fig. 1). Patients scored high on fish (optimum component, median intake 17.6 [7.8–31.8] g; median score 6.4 [IQR: 2.6–10], Fig. 2). Patients scored low on soy intake (adequacy component, median intake 0.0 [0.0–4.7] g, median score 0.0 [IQR 0.0–1.9], Fig. 2, Table S6), red and processed meats (moderation component, median intake 75.1 [42.2–115.6] gram; median score 0.0 [IQR: 0.0–0.0], Fig. 2, Table S6) and unsaturated oils (optimum component, median intake 3.8 [1.1–11.4] gram; median score 0.0 [IQR: 0.3–2.6], Fig. 2, Table S6).

### Relationship of the EAT-Lancet diet with CVD

During a median follow-up 9.13 years, 209 non-fatal vascular events occurred in 2553 patients. A diet more in line with the EAT-Lancet HRD was associated with a lower risk of non-fatal vascular events (HR 0.87 (95% CI 0.79–0.96) per 10-point increase and HR 0.63 (95% CI 0.44–0.91) 3rd vs. 1st tertile, Table 2). There was a trend towards a relationship with lower risk of myocardial infarction (HR 0.90 (95% CI 0.81–1.02) per 10-point increase and HR 0.69 (95% CI 0.45–1.06) 3rd vs. 1st tertile). For stroke, a diet more in line with the EAT-Lancet HRD was associated with a lower risk (HR 0.76 (95% CI 0.63–0.91) per 10-point increase, HR 0.46 (95% CI 0.23–0.88) 3rd vs. 1st tertile).

**Table 2:** The relationship between the EAT-Lancet Healthy Reference Diet and CVD

	Events	Follow-up (person-years)	EAT-Lancet HRD (HR (95% CI))			
			Per 10-point increase	1st tertile (N = 851)	2nd tertile (N = 851)	3rd tertile (N = 851)
Non-fatal vascular events	209	24,212				
Model 1			0.87 (0.79–0.95)	Reference	0.58 (0.42–0.81)	0.62 (0.44–0.87)
Model 2			0.87 (0.79–0.96)	Reference	0.58 (0.41–0.80)	0.63 (0.44–0.91)
Model 3			0.88 (0.79–0.97)	Reference	0.60 (0.43–0.83)	0.66 (0.46–0.94)
Non-fatal myocardial infarction	157	24,556				
Model 1			0.89 (0.80–0.98)	Reference	0.67 (0.46–0.96)	0.64 (0.43–0.95)
Model 2			0.90 (0.81–1.02)	Reference	0.68 (0.46–0.99)	0.69 (0.45–1.06)
Model 3			0.92 (0.81–1.03)	Reference	0.70 (0.48–1.03)	0.72 (0.47–1.09)
Non-fatal stroke	59	25,248				
Model 1			0.79 (0.67–0.94)	Reference	0.36 (0.19–0.70)	0.49 (0.26–0.91)
Model 2			0.76 (0.63–0.91)	Reference	0.35 (0.18–0.69)	0.46 (0.23–0.88)
Model 3			0.76 (0.63–0.92)	Reference	0.36 (0.18–0.71)	0.47 (0.24–0.92)

The relationship between the EAT-Lancet Healthy Reference Diet (HRD) and non-fatal CVD was assessed using three Cox regression models adjusted for the following confounders. Model 1: age and sex. Model 2: model 1 + level of education, physical activity, alcohol intake, smoking status, number of packyears, year of inclusion and energy intake (kilocalories). Model 3: model 2 + body mass index non-high-density-lipoprotein cholesterol, systolic blood pressure, glycated haemoglobin, estimated glomerular filtration rate, use of statins and number of antihypertensives. Results are expressed using the hazard ratio with corresponding 95% confidence interval

### Relationship of components of the Lancet-EAT diet with CVD

The following adequacy components, meaning a higher intake leads to a higher score, of the EAT-Lancet HRD score were associated with a lower risk of CVD: vegetables (HR 0.92 (95% CI 0.87–0.98) per point increase, Table 3), legumes (HR 0.92 (95% CI 0.86–0.99) per point increase) and soy (HR 0.92 (95% CI 0.87–0.98) per point increase, Table 3). None of the optimum components were related to CVD risk. There was no significant relationship between the moderation components on their own and CVD risk (Table 3). Overall, there was no evidence that one component was driving the relationship, as leaving out one of these 14 components at a time did not change the relationship between the EAT-Lancet HRD and risk of non-fatal vascular events (Table 3).

**Table 3:** The relationship of the separate food categories with CVD and the relationship of EAT-Lancet HRD Score with CVD after removal of one separate food category

Food group score	HR per point increase in score for independent food group	HR per 10-point increase in EAT-Lancet score after removal of food group
Main analysis	NA	0.87 (0.79–0.96)
Vegetables (0–10)	0.92 (0.87–0.98)	0.88 (0.79–0.98)
Fruit (0–10)	0.99 (0.95–1.03)	0.85 (0.76–0.95)
Grains (0–10)	0.98 (0.93–1.04)	0.87 (0.79–0.97)
Legumes (0–10)	0.92 (0.86–0.99)	0.88 (0.79–0.97)
Soy (0–10)	0.92 (0.87–0.98)	0.88 (0.79–0.98)
Red and processed meat (0–10)	0.96 (0.88–1.04)	0.88 (0.80–0.97)
Sugar from sweeteners (0–10)	0.97 (0.92–1.01)	0.88 (0.79–0.97)
Unsaturated fats (0–10)	0.98 (0.93–1.03)	0.87 (0.79–0.96)
Dairy (0–10)	0.98 (0.95–1.02)	0.86 (0.77–0.96)
Potatoes (0–10)	1.01 (0.97–1.05)	0.84 (0.76–0.94)
Meat (poultry) (0–10)	1.01 (0.97–1.05)	0.84 (0.75–0.94)
Eggs (0–10)	0.98 (0.95–1.02)	0.86 (0.77–0.96)
Fish (0–10)	0.99 (0.95–1.03)	0.85 (0.76–0.95)
Nuts (0–10)	0.98 (0.93–1.03)	0.87 (0.78–0.96)

The relationship of scores for 14 food groups making up the EAT-Lancet HRD with non-fatal CVD. The relationship between each food group and non-fatal CVD was modelled separately. Thereafter, the relationship of EAT-score with non-fatal CVD is displayed after the score for that food group was removed from the total EAT-Lancet score, resulting in a score ranging from 0 to 130. All models were adjusted for age, sex, level of education, physical activity, alcohol intake, smoking status, number of packyears, year of inclusion and energy intake (kilocalories). A description of each food group is provided in Table S2. HRD = Healthy Reference Diet. HR(95% CI) = Hazard ratio (95% confidence interval)

### Sensitivity analyses

Including only patients with a baseline visit within 9 years of the FFQ potentially increased the strength of the relationship, although with less precision (HR per 10-point increase for non-fatal vascular events 0.77 (95% CI 0.59–1.01), Table S3). Use of the energy-adjusted EAT-lancet score did not change the results (HR for non-fatal vascular events 0.87 (95% CI 0.79–0.96) Table S7). There was no evidence of effect modification in subgroups (Table S2).

## Discussion

In patients with established CVD, a diet more in line with the EAT-Lancet HRD was associated with a lower risk of non-fatal vascular events. There was an association with a lower risk of stroke and a trend towards a lower risk of myocardial infarction.

Results of the present study are in line with effect estimates in the general population, where the EAT-Lancet diet is associated with a lower risk of fatal and non-fatal CVD (HR Q1 vs. Q4: 0.86 (95% CI 0.78–0.94)) [5]. Another study reported a non-significant relationship (HR 0.85 (95% CI 0.62–1.16) per 100-point increase (range: − 162 to 332 points) [15]. However, these studies did not include patients with established CVD.

In the present study, a diet closer to the EAT-Lancet HRD was associated with lower risk of non-fatal stroke. One previous study in apparently healthy adults over 50 years old, also found that the EAT-Lancet diet was associated with a lower risk of ischemic stroke and intracerebral hemorrhage (HR 0.76 (95% CI 0.64–0.90) and HR 0.58 (95% CI 0.36–0.93)) [16]. However, that study used a score which was criticized for not appropriately scoring the EAT-Lancet diet, using a binary scoring system [17]. Another study using the same score did not find a relationship between the EAT-Lancet score and risk of stroke in the British general population (HR 1.06 (95% CI 0.87–1.28) [18]. Lastly, a study in the general population reported a trend towards a lower risk of fatal and non-fatal stroke (HR 0.89 (95% CI 0.72–1.10) [5]. The EAT-Lancet dietary pattern is low in sodium-rich products such as cured meat, fish products and processed cheeses and high in low-salt, unprocessed foods, such as fruits and vegetables. Therefore part of the relationship with stroke in the present study might be explained by a lower sodium intake. In line with this, in the present study SBP was 2 mmHg lower in Q3 vs Q1 of the Eat-Lancet HRD. This hypothesis can however not be confirmed given that sodium intake cannot be reliably estimated using FFQs [19].

Furthermore, in the present study there was a trend for a lower risk of non-fatal MI in patients adhering to the EAT-Lancet HRD. Previous studies in the general population did find a relation between the EAT-Lancet diet and incident coronary heart disease risk (HR 0.88 (95% CI 0.78–1.00) and HR 0.80 (95% CI 0.67–0.96) for high vs low adherence) [5, 20]. Whether the trend in the present study represents a weaker relationship in patients who are already using risk factor-lowering medication or whether the current results are due to a lack of power cannot be determined. However, given that the effect estimate is protective and the confidence interval is broad, a lack of power for this outcome seems likely.

The findings of the present study imply that patients with CVD might benefit from adopting the EAT-Lancet diet. There was no evidence of effect modification in patients who achieved treatment goals for CVD risk factors (SBP below compared to above140 mmHg or non-HDL-C below compared to above 2.6 mmol/L), which suggests that all patients with CVD might benefit, irrespective of achieved risk factor levels. In addition to health benefits, dietary interventions can reduce healthcare costs in a secondary prevention setting [21]. Modeling studies with plant-based diets show that health benefits can translate to reduced societal costs, such as reduced healthcare costs and costs of absenteeism [22]. However, given the observational nature of the current study, future randomized trials are needed to assess whether adopting the EAT-Lancet healthy reference diet, indeed lowers recurrent (cardio)vascular events in patients with CVD.

The present study demonstrates that the diet of CVD patients is currently not well aligned with the EAT-Lancet HRD. Especially high intake of red and processed meat and sugar, and low intake of soy, legumes and nuts, resulted in low scores. Reducing intake of sugar, red and processed meat and increasing intake of soy, legumes and nuts might therefore improve the CVD-risk profile of these patients. While the EAT-Lancet HRD is relatively new, recommendations to reduce for instance red and processed meat and sugar intake have been made consistently by guidelines [1, 2]. These findings imply that further implementation of these recommendations is needed. [23–25]Some strengths and limitations of this study should be considered. Strengths include the detailed assessment of dietary intake using a validated FFQ [8]. Additionally, the extensive collection of baseline characteristics allowed for extensive adjustment for confounders, although residual confounding in unmeasured variables cannot be excluded. The systematic collection of baseline characteristics and outcome variables in the UCC-SMART ensured low levels of missing data. Lastly, standardized assessment of outcomes by three physicians of outcome committees using hospital discharge letters likely reduced the chance of misclassification of outcomes in the current study. Limitations include the timing of the FFQ, which was conducted retrospectively in all alive UCC-SMART participants in 2022. Therefore no temporality can be established, which is one of the Bradford-Hill criteria for inferring causality in observational research [23]. However, previous studies have already established a temporal relationship between dietary intake and CVD events in other populations [5, 16, 20]. In this observational study, analyses were adjusted for confounding factors but residual confounding may affect the relation between Lancet-EAT and risk of recurrent CVD events. Secondly, the collection of the FFQ after baseline might have also resulted in survivor bias. Patients who experienced a non-fatal event might have adopted a healthier dietary pattern, which might have attenuated the results. In general, people in the Netherlands have shifted to a diet lower in red and processed meats and higher in plant-based foods such as legumes over the last two decades [24]. Given that diet was assessed in 2022, patients might have been reporting a diet higher in plant-based foods, and therefore closer to the EAT-Lancet recommendations, than the diet they had been consuming the years prior. This might have led to an underestimation of the relationship. Similarly, due to healthy survivor bias, alignment with the EAT-Lancet might be overestimated, given that patients who completed the FFQ were more often non-smokers with a high level of education. Additionally, it should be noted that while the EAT-Lancet HRD overlaps with several CVD guideline recommendations, some specific recommendations aimed at reducing CVD are not included in the HRD, such as minimizing saturated fat intake, trans-fat intake and reducing salt intake below 5 g/d [1, 2]. These dietary factors remain important for CVD prevention and should be considered when counselling patients with established CVD. [25–28] Lastly, the FFQ used to assess dietary patterns, did not collect data on whole-grain intake from pasta or rice. Whole-grain intake might therefore be underestimated. However, the majority of whole-grain intake is the result of intake from bread and cereals, which was assessed with the present FFQ. The reported whole-grain intake in the present study (82.9 g) is only modestly lower than the 93 to 101 g reported in another study in the Dutch population [29]. The findings of the present study therefore need to be confirmed in large observational cohort studies and ideally in a randomized controlled intervention study before large-scale implementation of EAT-Lancet HRD in clinical practice.

In conclusion, the EAT-Lancet diet is associated with a lower risk of recurrent (cardio)vascular events in patients with established CVD. Clinicians should note that while the EAT-Lancet diet aligns with many CVD guideline recommendations, additional specific focus on saturated fat, trans-fat and salt intake remains warranted during dietary counselling.

## Supplementary Information

Below is the link to the electronic supplementary material.

Supplementary file1 (DOCX 300 kb)
