# Supplementary material for: The relationship between the EAT-Lancet dietary pattern and risk of cardiovascular events in patients with established cardiovascular disease
Source: Eur J Nutr. 2025 Dec 1;64(8):324. doi: 10.1007/s00394-025-03754-2 (PMC12669304; doi:10.1007/s00394-025-03754-2)
Supplement: Supplementary file 1 — Supplementary file1 (DOCX 300 kb) [file 394_2025_3754_MOESM1_ESM.docx]

**Supplemental tables and figures**

**Figure S1.** Overview of patients included in the main analysis.


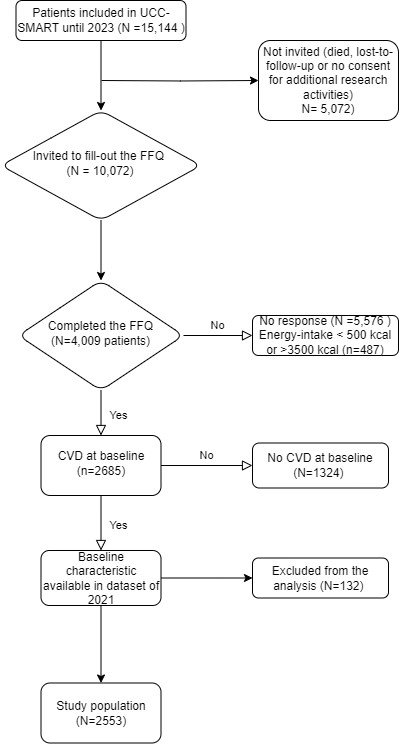


CVD = cardiovascular disease. SMART = Utrecht Cardiovasculair Cohort Second Manifestations of ARTerial diseases. FFQ = food frequency questionnaire. Kcal = kilocalorie.

**Table S1**. Calculation of the EAT-Lancet HRD score using the sum of the scores for the 14 food groups, based on Colizzi et al^2^

| Food group | Classification | Explanation of included foods | Recommended intake  (g/day) | Dietary intake and the corresponding score  (grams per day) | | | |
| --- | --- | --- | --- | --- | --- | --- | --- |
|  |  |  |  | 0 points | Proportional score from 0 to 10 | 10 points | Proportional score from 10 to 0 |
| Whole grains | Adequacy | Brown, multigrain and rye bread, bread, whole wheat bread, and whole wheat rolls. Data on whole grain rice and pasta is not available. | 464 | 0 | 0-464 | ≥464 |  |
| Vegetables | Adequacy | Cruciferous Vegetables (e.g. cauliflower). Root Vegetables (e.g. carrots). Leafy Greens (e.g. spinach). Other Vegetables: Tomatoes, cucumber, and various other cooked, stir-fried or fermented vegetables. | 300 | 0 | 0-300 | ≥300 |  |
| Fruits | Adequacy | Citrus fruits (e.g. oranges). Pome Fruits (e.g. apples). Tropical fruits (e.g. bananas). Berries (e.g. grapes). | 200 | 0 | 0-200 | ≥200 |  |
| Legumes | Adequacy | Legumes such as kidney beans and lentils. Peas and broad beans were considered vegetables. | 50 | 0 | 0-50 | ≥50 |  |
| Soy | Adequacy | Soy drink and yoghurt, tempeh and vegetarian meat replacements | 25 | 0 | 0-25 | ≥25 |  |
| Potatoes | Optimum | Cooked, mashed, baked or fried potatoes. | 50 | 0 | 0-50 | 50-100 | 100-150 |
| Dairy | Optimum | Cheeses, milk, yoghurts, cream(s), coffee milk. | 250 | 0 | 0-250 | 250-500 | 500-750 |
| Poultry | Optimum | Chicken meats, skinned and not skinned | 29 | 0 | 0-29 | 29-58 | 58-88 |
| Eggs | Optimum | Whole eggs | 13 | 0 | 0-13 | 13-25 | 25-38 |
| Fish | Optimum | Lean fish (e.g. pangasius), fatty fish (e.g.salmon), battered fish (e.g. fish sticks), smoked fish (e.g. salmon), seafood (e.g.shrimp). | 28 | 0 | 0-28 | 28-100 | 100-128 |
| Nuts | Optimum | Nuts, seeds, peanut butter | 50 | 0 | 0-50 | 50-100 | 100-150 |
| Unsaturated oils | Optimum | Intake from unsaturated oils was the sum of intake of olive oil, liquid baking products and margarine | 40 | 0 | 0-40 | 40-80 | 80-120 |
| Red meat (Beef, lamb and pork) | Moderation | Beef and pork mince, hamburgers, various meats and game, smoked sausages, bacon, cold cuts, liver-based products, and various sausages | 14 | ≥14 | 14-0 | 0 |  |
| Sugar from sweeteners | Moderation | Sucrose/saccharose and monosaccharides from the following food groups. Sugar and sweets such as candy, cookies, cakes and biscuits. Sugary soft drinks such as soft drinks, lemonades, sports drinks and energy drinks. Fruit juices such as apple juice and orange juice. | 31 | ≥31 | 31-0 | 0 |  |

The EAT-Lancet HRD score was the sum of the scores for all 14 food groups, based on Colizzi et al^2^. The maximum score for each food category is 10, resulting in a maximum score of 140. For whole grains, vegetables, fruits, legumes and soy, a higher intake resulted in a higher score for that food group. Potatoes, dairy, chicken, eggs, fish, nuts and unsaturated oils were scored based on an optimum. An intake in the optimum range resulted in the maximum score for that food group (10 points) and an intake below or above this optimum resulted in a lower score. A higher intake of red meat and sugar from sweeteners resulted in a lower score for those food groups. Amounts are provided in grams and are the amounts for men. Amounts were adopted for women using a caloric intake of 2000 instead 2500 kcal/day. References above were divided by 2500 and multiplied by 2000 to obtain the reference values for women, as was done in article by Colizzi et al.^2^

**Table S2** The relationship between the EAT-Lancet Healthy Reference Diet and non-fatal CVD in subgroups


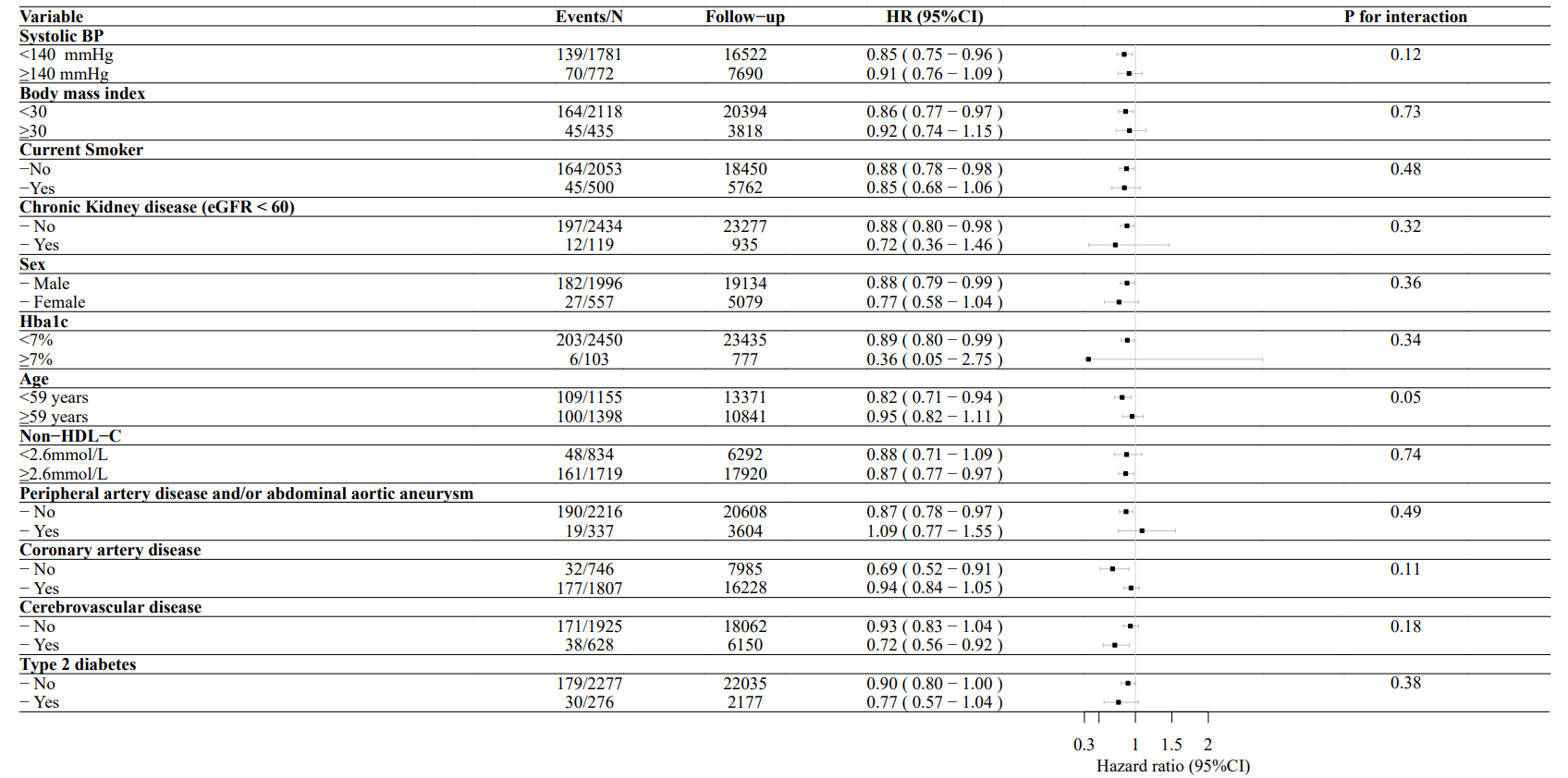


The relationship between the EAT-Lancet Healthy Reference Diet and non-fatal CVD was quantified using a Cox model and adjusted for age, sex, level of education, physical activity, alcohol intake, smoking status, number of packyears, year of inclusion and energy intake (kilocalories), body mass index, non-high-density-lipoprotein cholesterol (non-HDL-c), systolic blood pressure, glycated haemoglobin (Hba1c), estimated glomerular filtration rate, use of statins and number of antihypertensives (model 3). A likelihood ratio test was performed to test if the inclusion of an interaction term significantly improved the model fit, thus indicating a statistically significant interaction effect. Using Bonferroni correction for multiple testing, P < 0.0042 (p = 0.05 divided by 12 subgroups) should be considered significant. A non-HDL-c of 2.6 mmol/L corresponds to a low-density lipoprotein cholesterol of 1.8 mmol/L.^3^ HR (95%CI) = Hazard ratio (95% confidence interval) per 10 point increase.

|  | Events | Follow-up (person-years) | HR (95%CI) per 10-point increase |
| --- | --- | --- | --- |
| Non-fatal vascular events | 27 | 3893 |  |
| Model 1 |  |  | 0.75 (0.59-0.95) |
| Model 2 |  |  | 0.77 (0.59-1.01) |
| Model 3 |  |  | 0.80 (0.61-1.05) |

**Table S3**. Relationship between the EAT-Lancet Healthy Reference Diet and risk of vascular events in patients with a baseline visit within 9 years of the FFQ.

The relationship between the EAT-Lancet Healthy Reference Diet and non-fatal CVD in patients with a baseline visit within 9 years of the FFQ collection. Model 1 was adjusted for age and sex. Model 2 for model 1 + level of education, physical activity, alcohol intake, smoking status, number of packyears, year of inclusion and energy intake (kilocalories). Model 3 was adjusted for: model 2 + body mass index, non-high-density-lipoprotein cholesterol, systolic blood pressure, glycated haemoglobin, estimated glomerular filtration rate, use of statins and number of antihypertensives. FFQ = food frequency questionnaire. HR[95%CI) = Hazard ratio (95% confidence interval)

**Table S4** Baseline characteristics stratified in tertiles of the energy-adjusted EAT-Lancet Score

|  | Overall | Energy-adjusted  EAT-Lancet score | | | | | | |
| --- | --- | --- | --- | --- | --- | --- | --- | --- |
|  | (N=2,553) | 1^st^ tertile  (N=851) | | | 2^nd^ tertile  (N=851) | | | 3rd tertile  (N=851) |
| Age (years) | 59 ±9 | 59 ±10 | | | 59 ±9 | | | 59 ±9 |
| Sex (female) | 557 (22) | 148 (17) | | | 186 (22) | | | 223 (26) |
| Level of education |  | | |  | | |  | |
| Low | 479 (19) | 204 (24) | | | 170 (20) | | | 105 (12) |
| Medium | 1006 (39) | 363 (43) | | | 329 (39) | | | 314 (37) |
| High | 1068 (42) | 284 (33) | | | 352 (41) | | | 432 (51) |
| Alcohol consumption (units/week) | | | |  | | |  | |
| 0 – 10 | 1893 (74) | 622 (73) | | | 631 (74) | | | 640 (75) |
| 11-20 | 463 (18) | 153 (18) | | | 163 (19) | | | 147 (17) |
| >20 | 197 (8) | 76 (9) | | | 57 (7) | | | 64 (8) |
| Smoking Status |  | | |  | | |  | |
| Never | 743 (29) | 216 (25) | | | 242 (28) | | | 285 (34) |
| Current | 500 (20) | 204 (24) | | | 179 (21) | | | 117 (14) |
| Former | 1310 (51) | 431 (51) | | | 430 (51) | | | 449 (53) |
| Packyears | 9 [0-24] | 12 [0-28] | | | 9 [0-24] | | | 6 [0-20] |
| Physical activity (METh/week) | | | |  | | |  | |
| 1st tertile | 851 (33) | 313 (37) | | | 290 (34) | | | 248 (29) |
| 2nd tertile | 851 (33) | 271 (32) | | | 286 (34) | | | 294 (35) |
| 3rd tertile | 851 (33) | 267 (31) | | | 275 (32) | | | 309 (36) |
| History of CVD |  | |  | | |  | |  |
| CAD | 1625 (64) | 509 (60) | | | 546 (64) | | | 570 (67) |
| CeVD | 502 (20) | 181 (21) | | | 165 (19) | | | 156 (18) |
| PAD and/or AAA | 211 (8) | 78 (9) | | | 69 (8) | | | 64 (8) |
| >1 location | 215 (8) | 83 (10) | | | 71 (8) | | | 61 (7) |
| Hypertension | 1321 (52) | 425 (50) | | | 445 (52) | | | 451 (53) |
| Type 2 diabetes | 276 (11) | 93 (11) | | | 94 (11) | | | 89 (11) |
| BMI (kg/m^2^) | 26.7 ±3.9 | 27.1 ±3.9 | | | 26.8 ±4.3 | | | 26.4 ±3.6 |
| SBP (mmHg) | 134 ±18 | 135 ±19 | | | 133 ±17 | | | 133 ±18 |
| HbA1c (%) | 5.6 [5.4-5.8] | 5.6 [5.4-5.9] | | | 5.5 [5.4-5.8] | | | 5.5 [5.4-5.8] |
| Non-HDL-c (mmol/L) | 3.0 [2.5-3.8] | 3.1 [2.5-3.9] | | | 3.0 [2.5-3.8] | | | 2.9 [2.4-3.6] |
| eGFR (mL/min/1.73m^2^) | 91 [78-100] | 88 [76-99] | | | 91 [79-100] | | | 93 [82-102] |
| C-reactive protein (mg/L) | 1.5 [0.8-3.1] | 1.6 [0.9-3.4] | | | 1.5 [0.8-3.0] | | | 1.5 [0.8-3.2] |
| NLR | 1.9 [1.5-2.5] | 1.9 [1.5-2.6] | | | 1.9 [1.5-2.5] | | | 1.9 [1.5-2.5] |
| Number of anti-hypertensives | 2.5 (1.1) | 2.5 (1.1) | | | 2.5 (1.1) | | | 2.5 (1.1) |
| Statin prescribed (%) | 2014 (79) | 650 (76) | | | 668 (79) | | | 696 (82) |

Baseline characteristics, stratified by adherence to the EAT-Lancet Healthy Reference Diet after energy-adjustment using the residuals method.^4^ Patient characteristics are presented as mean (standard deviation), median [interquartile range] or count (percentage). CVD = (cardio)vascular disease. CeVD = cerebrovascular disease. PAD = peripheral artery Disease. AAA = abdominal aortic aneurysm. BMI = body mass index. SBP = systolic blood pressure. Non-HDL-c = non-high-density lipoprotein cholesterol. eGFR = estimated glomerular filtration rate calculated using the 2021 creatinine-based equations^1^. NLR = neutrophil-to-lymphocyte ratio.

**Table S5.** Dietary intake in tertiles of the EAT-Lancet Healthy Reference Diet score

|  | Overall | EAT-Lancet score | | |
| --- | --- | --- | --- | --- |
|  | (N=2,553) | 1^st^ tertile  (N=851) | 2^nd^ tertile  (N=851) | 3rd tertile  (N=851) |
| Fruit (grams) | 115.2 [41.1, 229.2] | 50.6 [2.4, 122.2] | 116.8 [54.7, 225.1] | 214.5 [111.1, 251.3] |
| Vegetables (grams) | 105.3 [54.9, 166.2] | 64.1 [19.3, 113.0] | 105.3 [62.9, 153.2] | 151.7 [99.5, 219.6] |
| Whole grains (grams) | 82.9 [49.2, 138.0] | 70.5 [27.6, 111.3] | 80.5 [49.3, 134.9] | 105.8 [70.0, 147.9] |
| Potatoes (grams) | 61.5 [30.0, 101.0] | 55.7 [19.0, 113.9] | 64.0 [32.0, 101.0] | 63.0 [40.8, 92.5] |
| Red and processed meat (grams) | 75.1 [42.2, 115.6] | 71.0 [38.4, 115.2] | 80.3 [48.2, 118.9] | 72.2 [40.0, 111.2] |
| Poultry (grams) | 17.5 [7.0, 36.2] | 10.5 [2.1, 21.6] | 17.7 [10.5, 36.4] | 21.8 [13.7, 42.8] |
| Dairy (grams) | 270.5 [140.5, 429.5] | 194.9 [59.6, 411.7] | 281.9 [157.6, 428.2] | 315.3 [202.8, 439.3] |
| Eggs (grams) | 14.0 [7.0, 21.5] | 9.0 [4.5, 29.0] | 14.0 [7.0, 21.5] | 14.5 [7.0, 21.5] |
| Fish (grams) | 17.6 [7.8, 31.8] | 9.0 [2.0, 17.5] | 18.5 [10.5, 31.7] | 27.5 [17.5, 42.0] |
| Soy (grams) | 0.0 [0.0, 4.7] | 0.0 [0.0, 0.0] | 0.0 [0.0, 3.3] | 3.5 [0.0, 18.8] |
| Nuts (grams) | 8.9 [2.5, 21.8] | 3.7 [0.8, 11.8] | 8.1 [2.9, 18.5] | 18.4 [8.2, 31.9] |
| Legumes (dry weight in grams) | 6.8 [2.9, 14.0] | 4.4 [1.0, 10.2] | 6.8 [2.9, 14.0] | 10.4 [6.5, 21.0] |
| Unsaturated oils (grams) | 3.8 [1.1, 11.4] | 1.9 [0.3, 6.5] | 3.7 [1.2, 11.2] | 6.0 [2.7, 17.8] |
| Sugar from sweeteners (grams) | 20.5 [9.6, 36.4] | 22.9 [8.4, 40.7] | 22.0 [11.7, 36.9] | 17.6 [9.2, 29.6] |
| Sugar, except sugar from fruit (grams) | 61.7 [42.4, 86.9] | 62.4 [31.9, 89.5] | 62.5 [43.1, 88.8] | 60.4 [46.0, 82.4] |
| Energy-intake (kilocalorie) | 1913.0 [1513.0, 2349.6] | 1688.0 [1176.3, 2153.9] | 1922.0 [1543.0, 2328.4] | 2106.1 [1735.6, 2511.8] |

Dietary intake in tertiles of the EAT-Lancet HRD score. A complete description of the food categories is provided in table S2. In short, fruit includes all fresh fruits, vegetables includes all vegetables except legumes. Whole-grains includes all sources of whole-grain except from pasta and rice. Potatoes includes potatoes (cooked, mashed, fried or baked). Red and processed meats includes all meats from beef, lamb and pork. Poultry includes chicken (skinned and not-skinned). Dairy includes all types of dairy (cheese, milk, yoghurts). Eggs were whole eggs. Fish included all fish types and seafood. Soy included intake of soy milk, tempeh, vegetarian meat replacements and other soy products. Nuts included all nut types and peanuts. Legumes includes legumes except peas and broad beans which were considered vegetables. Unsaturated oils were all added unsaturated oils during food preparation. Added sugar included sugar from bakery products, soft drinks, fruit juices and candy.

**Table S6.** Dietary intake in tertiles of the energy-adjusted EAT-Lancet Healthy Reference Diet score

|  | Overall | EAT-Lancet score | | |
| --- | --- | --- | --- | --- |
|  | (N=2,553) | 1^st^ tertile  (N=851) | 2^nd^ tertile  (N=851) | 3rd tertile  (N=851) |
| Fruit (grams) | 115.2 [41.1, 229.2] | 55.5 [4.5, 139.1] | 112.2 [51.2, 221.6] | 209.0 [109.9, 247.7] |
| Vegetables (grams) | 105.3 [54.9, 166.2] | 71.0 [21.3, 123.3] | 103.2 [61.2, 157.0] | 146.1 [93.2, 215.6] |
| Whole grains (grams) | 82.9 [49.2, 138.0] | 74.9 [35.2, 133.3] | 79.1 [45.1, 138.0] | 92.0 [62.5, 140.7] |
| Potatoes (grams) | 61.5 [30.0, 101.0] | 73.6 [23.5, 124.1] | 61.0 [30.0, 97.4] | 57.1 [35.5, 84.6] |
| Red and processed meat (grams) | 75.1 [42.2, 115.6] | 86.2 [45.4, 132.5] | 78.3 [47.5, 114.1] | 64.9 [35.5, 100.8] |
| Poultry (grams) | 17.5 [7.0, 36.2] | 11.2 [2.5, 28.9] | 17.7 [10.2, 36.5] | 21.2 [11.2, 36.4] |
| Dairy (grams) | 270.5 [140.5, 429.5] | 225.3 [80.0, 473.2] | 270.5 [152.3, 423.0] | 290.6 [182.4, 420.0] |
| Eggs (grams) | 14.0 [7.0, 21.5] | 14.0 [4.5, 29.0] | 14.0 [7.0, 21.5] | 14.5 [7.0, 21.5] |
| Fish (grams) | 17.6 [7.8, 31.8] | 9.2 [2.0, 18.7] | 18.4 [9.5, 31.8] | 25.8 [16.4, 39.5] |
| Soy (grams) | 0.0 [0.0, 4.7] | 0.0 [0.0, 0.0] | 0.0 [0.0, 3.5] | 3.6 [0.0, 18.9] |
| Nuts (grams) | 8.9 [2.5, 21.8] | 4.6 [0.8, 14.0] | 8.5 [3.0, 20.1] | 16.8 [5.8, 29.7] |
| Legumes (dry weight in grams) | 6.8 [2.9, 14.0] | 4.4 [1.0, 10.2] | 6.8 [3.4, 14.0] | 10.2 [6.5, 21.0] |
| Unsaturated oils (grams) | 3.8 [1.1, 11.4] | 2.3 [0.4, 7.2] | 3.8 [1.2, 11.1] | 5.7 [2.3, 15.1] |
| Sugar from sweeteners (grams) | 20.5 [9.6, 36.4] | 28.1 [12.2, 46.2] | 21.7 [11.8, 35.3] | 14.6 [7.8, 24.8] |
| Sugar, except sugar from fruit (grams) | 61.7 [42.4, 86.9] | 72.0 [43.9, 102.3] | 63.0 [43.3, 85.8] | 54.8 [41.3, 74.8] |
| Energy-intake (kilocalorie) | 1913.0 [1513.0, 2349.6] | 1973.5 [1442.1, 2476.7] | 1900.6 [1481.0, 2310.7] | 1889.1 [1555.1, 2289.1] |

Dietary intake in tertiles of the EAT-Lancet HRD score after energy-adjustment using the residuals method. A complete description of the food categories is provided in table S2. In short, fruit includes all fresh fruits, vegetables includes all vegetables except legumes. Whole-grains includes all sources of whole-grain except from pasta and rice. Potatoes includes potatoes (cooked, mashed, fried or baked). Red and processed meats includes all meats from beef, lamb and pork. Poultry includes chicken (skinned and not-skinned). Dairy includes all types of dairy (cheese, milk, yoghurts). Eggs were whole eggs. Fish included all fish types and seafood. Soy included intake of soy milk, tempeh, vegetarian meat replacements and other soy products. Nuts included all nut types and peanuts. Legumes includes legumes except peas and broad beans which were considered vegetables. Unsaturated oils were all added unsaturated oils during food preparation. Sugar from sweeteners included sugar from bakery products, soft drinks, fruit juices and candy.

**Table S7.** The relationship of the EAT-Lancet Healthy Reference Diet with non-fatal vascular events, using the residuals method

|  | Events | Follow-up (person-years) | EAT-Lancet HRD  (HR (95% CI) per 10-point increase) |
| --- | --- | --- | --- |
| Non-fatal vascular events | 209 | 24,212 |  |
| Model 1 |  |  | 0.86 (0.78-0.95) |
| Model 2 |  |  | 0.87 (0.79-0.96) |
| Model 3 |  |  | 0.88 (0.79-0.97) |
| Non-fatal myocardial infarction | 157 | 24,556 |  |
| Model 1 |  |  | 0.90 (0.80-1.01) |
| Model 2 |  |  | 0.90 (0.81-1.02) |
| Model 3 |  |  | 0.92 (0.81-1.03) |
| Non-fatal Stroke | 59 | 25,248 |  |
| Model 1 |  |  | 0.74 (0.62-0.89) |
| Model 2 |  |  | 0.76 (0.63-0.91) |
| Model 3 |  |  | 0.76 (0.63-0.92) |

The relationship between the energy-adjusted EAT-Lancet score and non-fatal CVD. The EAT-Lancet Healthy Reference Diet score was adjusted for energy intake using the residuals method.^4^ Model 1 was adjusted for age and sex. Model 2 for model 1 + level of education, physical activity, alcohol intake, smoking status, number of packyears, year of inclusion and energy intake (kilocalories). Model 3 was adjusted for: model 2 + body mass index, non-high-density-lipoprotein cholesterol, systolic blood pressure, glycated haemoglobin, estimated glomerular filtration rate, use of statins and number of antihypertensives. FFQ = food frequency questionnaire. HR(95%CI) = Hazard ratio (95% confidence interval). HRD = Healthy Reference Diet.

|  |  | CVD patients with FFQ available ( n=2,553) | | | CVD patients with FFQ not available ( n=4,182) | | | | |
| --- | --- | --- | --- | --- | --- | --- | --- | --- | --- |
| Age (years) |  | 59 ±9 | | | 58 ±11 | | | | |
| Sex (female) |  | 557 (22) | | | | 1331 (32) | | | |
| Level of education |  | | |  | | | | |  |
| Low |  | 479 (19) | | | | 1252 (30) | | | |
| Medium |  | 1006 (39) | | | | 1856 (44) | | | |
| High |  | 1068 (42) | | | | 1074 (26) | | | |
| Alcohol consumption (units/week) | | | |  | | | | |  |
| 0 – 10 |  | 1893 (74) | | | | 3090 (74) | | | |
| 11-20 |  | 463 (18) | | | | 684 (16) | | | |
| >20 |  | 197 (8) | | | | 408 (10) | | | |
| Smoking Status |  | | |  | | | | |  |
| Never |  | 743 (29) | | | | 1015 (24) | | | |
| Current |  | 500 (20) | | | | 1321 (32) | | | |
| Former |  | 1310 (51) | | | | 1846 (44) | | | |
| Packyears |  | 9 [0-24] | | | | 13 [1, 30] | | | |
| Physical activity (METh/week) | | | |  | | | | |  |
| 1st tertile |  | 851 (33) | | | | 1261 (30) | | | |
| 2nd tertile |  | 851 (33) | | | | 1410 (34) | | | |
| 3rd tertile |  | 851 (33) | | | | 1511 (36) | | | |
| History of CVD |  | |  | | | |  |  | |
| CAD |  | 1625 (64) | | | | 2183 (52) | | | |
| CeVD |  | 502 (20) | | | | 986 (24) | | | |
| PAD and/or AAA |  | 211 (8) | | | | 548 (13) | | | |
| >1 location |  | 215 (8) | | | | 465 (11) | | | |
| Hypertension |  | 1321 (52) | | | | 2267 (54) | | | |
| Type 2 diabetes |  | 276 (11) | | | | 654 (16) | | | |
| BMI (kg/m^2^) |  | 26.7 ±3.9 | | | | 27.2 ±4 | | | |
| SBP (mmHg) |  | 134 ±18 | | | | 138 ±20 | | | |
| Glycated hemoglobin (%) |  | 5.6 [5.4-5.8] | | | | 5.6 [5.4-6.0] | | | |
| Non-HDL-c (mmol/L) |  | 3.0 [2.5-3.8] | | | | 3.3 [2.6-4.2] | | | |
| eGFR (mL/min/1.73m^2^) |  | 91 [78-100] | | | | 92 [78-102] | | | |
| C-reactive protein (mg/L) |  | 1.5 [0.8-3.1] | | | | 1.9 [0.9-4.0] | | | |
| Statin usage (%) |  | 2014 (79) | | | | 3001 (72) | | | |

**Table S8.** Baseline characteristics of CVD patients with and without FFQ data available

Baseline characteristics of CVD patients stratified by the presence of the food frequency questionnaire. Patients who did not complete the FFQ, did either not respond to the invitation or could not be invited because they did not provide consent for additional follow-up. Patients with an FFQ available are patients who responded to the FFQ (N= 2685) and with a plausible energy-intake between 500 and 3500 kilocallories (n= 2,553 patients, Figure S1). Characteristic are displayed as mean ± standard deviation, median [25th – 75th percentile] of absolute number (percentage). CVD = (cardio)vascular disease. CeVD = cerebrovascular disease. PAD = peripheral artery Disease. AAA = abdominal aortic aneurysm. BMI = body mass index. SBP = systolic blood pressure. Non-HDL-c = non-high-density lipoprotein cholesterol. eGFR = estimated glomerular filtration rate calculated using the 2021 creatinine-based equations^1^.

**Supplemental references**

1. Inker, L. A. *et al.* New Creatinine- and Cystatin C–Based Equations to Estimate GFR without Race. *New England Journal of Medicine* **385**, 1737–1749 (2021).

2. Colizzi, C. *et al.* Adherence to the EAT-Lancet Healthy Reference Diet in Relation to Risk of Cardiovascular Events and Environmental Impact: Results From the EPIC-NL Cohort. *J Am Heart Assoc* **12**, (2023).

3. Visseren, F. L. J. *et al.* 2021 ESC Guidelines on cardiovascular disease prevention in clinical practice. *European Heart Journal* vol. 42 3227–3337 Preprint at https://doi.org/10.1093/eurheartj/ehab484 (2021).

4. Tomova, G. D., Arnold, K. F., Gilthorpe, M. S. & Tennant, P. W. G. Adjustment for energy intake in nutritional research: a causal inference perspective. *American Journal of Clinical Nutrition* **115**, 189–198 (2022).
